# Automatic Bayesian single molecule identification for localization microscopy

**DOI:** 10.1038/srep33521

**Published:** 2016-09-19

**Authors:** Yunqing Tang, Johnny Hendriks, Thomas Gensch, Luru Dai, Junbai Li

**Affiliations:** 1 National Center for Nanoscience and Technology of China, Beijing 100190, P.R. China; 2 Institute of Complex Systems (ICS-4, Cellular Biophysics), Forschungszentrum Jülich, Jülich 52428, Germany; 3 Institute of Chemistry, Chinese Academy of Sciences, Beijing 100190, P.R. China

## Abstract

Single molecule localization microscopy (SMLM) is on its way to become a mainstream imaging technique in the life sciences. However, analysis of SMLM data is biased by user provided subjective parameters required by the analysis software. To remove this human bias we introduce here the Auto-Bayes method that executes the analysis of SMLM data automatically. We demonstrate the success of the method using the photoelectron count of an emitter as selection characteristic. Moreover, the principle can be used for any characteristic that is bimodally distributed with respect to false and true emitters. The method also allows generation of an emitter reliability map for estimating quality of SMLM-based structures. The potential of the Auto-Bayes method is shown by the fact that our first basic implementation was able to outperform all software packages that were compared in the ISBI online challenge in 2015, with respect to molecule detection (Jaccard index).

Single molecule localization microscopy (SMLM) techniques, such as photoactivated localization microscopy (PALM)^1^, fluorescence photo-activation localization microscopy (FPALM)^2^, stochastic optical reconstruction microscopy (STORM)^3^ and direct stochastic optical reconstruction microscopy (*d*STORM)^4^, can overcome the inherent resolution limit encountered in far field optical microscopy and provide nanometer scale resolution^5^. It has become widely popular in recent years and is one of the fluorescence super-resolution methods recognized by the Nobel Prize in Chemistry 2014^6^. A large number of SMLM analysis software packages are available by now^5^,^7^. In all packages, it is necessary to define characteristics of a true emitter in order to distinguish single emitter signals from background fluctuations. A variety of characteristics have been used such as signal-to-noise ratio (SNR)^8^,^9^,^10^,^11^,^12^, spot brightness^12^,^13^,^14^ and point spread function (PSF) size^11^,^13^. Depending on the software, these characteristics can be calculated using a variety of methods, like wavelet filtering^9^,^10^, Gaussian filtering^8^,^10^ or median filtering^10^,^15^. An analysis program may hardcode certain thresholds, but usually allows the user to choose their own thresholds, either directly or indirectly. In any case, it always requires the user to make the correct choice, a choice that even for expert users with the appropriate expertise can be difficult and time consuming^7^ since the reliability and quality of found emitters are heavily influenced by the chosen thresholds of these characteristics. Using wrong thresholds can lead to underestimation or overestimation of the number of true single molecule emitters and can lead to artificial loss or strengthening of fine details in the super-resolved structures. Therefore, a well-defined method to determine thresholds and a fully automated analysis tool are desirable, especially for the growing community of biophysicists, chemists, biologists, and medical scientists, who do not develop SMLM methods and analysis software but apply it to their research subjects.

In general, a typical analysis starts by determining all potential emitters in an image frame. The majority of the current SMLM software packages perform a pre-selection of these potential emitters, which is generally based on either user-modifiable or hardcoded thresholds. Non-optimal pre-selection thresholds can result in low identification accuracy. In contrast, we found if no pre-selection is applied and all candidates (locally brightest spots) are fitted with a PSF, the photoelectron counts present a bimodal distribution corresponding to false and true single molecule emitters, respectively. This feature reflects the fact that a true emitter contributes more photoelectrons compared with background fluctuations. Naturally, the threshold for which the identification error is minimal localizes in the saddle between the two peaks of the bimodal photoelectron counts distribution. This feature offers the possibility to determine the optimized threshold automatically using a Bayesian statistics based method that uses two *a priori* distributions, one for false and one for true emitters. Based on this strategy, we introduce Auto-Bayes, a fully automated single molecule identification and localization analysis method. Auto-Bayes requires only objective parameters that are merely determined by the experimental conditions. No arbitrary input or tuning of parameters is necessary. An implementation of the Auto-Bayes method took part in the ISBI permanent online challenge for single molecule localization microscopy software^7^ in 2015 and achieved excellent and robust performance (see Table 1). Notably, Bayesian statistics has been used to solve other localization microscopy related problems as well, *e.g.*, in the analysis of high-density fluorophore images^16^ and the evaluation of molecular cluster assignment proposals^17^.

A SMLM image is reconstructed based on localization of individual single molecule emitters. No localization algorithm can, however, completely avoid the occurrence of false positive and false negative emitters. The reliability of a super-resolved structure is therefore determined by the ‘is a true emitter’-probability for each emitter it contains. This greatly influences the confidence of the conclusions that are based upon such a super-resolved structure. Since the Auto-Bayes method estimates the distribution of both false and true emitters, it is possible to derive the reliability of an emitter. Accordingly, a reliability map of a super-resolution image can be made that offers an alternative point of view.

Furthermore, we found that if the loosest acceptance condition (*i.e.*, no or weakest pre-selection) is used in several other SMLM software packages, the distributions of the corresponding characteristics are also bimodal in nature. In such cases, the Bayesian statistics method can also be applied if suitable *a priori* distributions are supposed for false and true emitters. Therefore, adding this capability to those software packages would allow automatic single molecule identification and make them more usable for both non-expert and expert users.

## Results

To evaluate SMLM analysis methods, Sage *et al*.^7^ have created simulated reference datasets and provided them to the community as part of a permanent challenge. Similar to a real experiment, the actual positions of the emitters that were used to generate the datasets are not known to us. In addition three training datasets are available that were generated in the same way. For these, however, the actual positions of the emitters are also provided. We first demonstrate the principle of our method by using the training datasets, as they allow us to categorize each analyzed locally brightest spot as either a false or true emitter.

**Figure 1:**
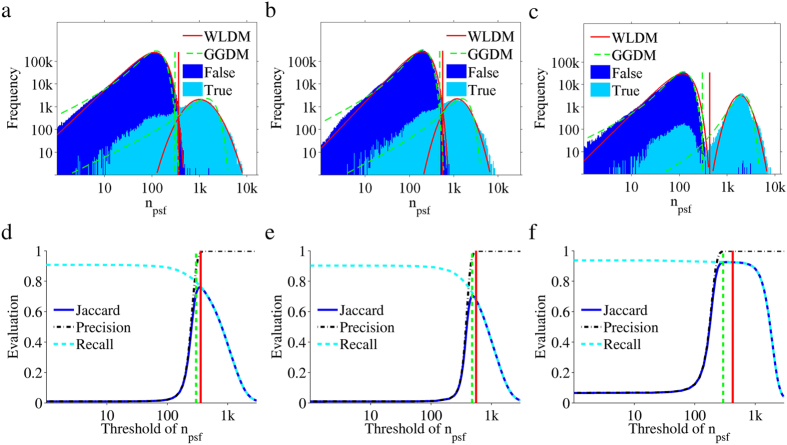
Auto-Bayes analysis for three training datasets. (**a–c**) *n*_*psf*_ distribution with GMET analysis for the training datasets Tubulins I, Tubulins II and Bundle of Tubulins, respectively. Three evaluation indexes (Jaccard, Recall and Precision^7^; see Methods) are employed to evaluate the results. Recall represents the recovery of true emitters and Precision represents the fraction of true emitter. Jaccard both counts for Recall and Precision and is the most relevant index. The highest Jaccard corresponds to an optimized threshold. Jaccard, Precision and Recall plotted as a function of the *n*_*psf*_ threshold are shown for datasets Tubulins I, Tubulins II and Bundles of Tubulins in (**d–f**), respectively. The *n*_*psf*_ thresholds are 356.5, 556.5, and 424.5 respectively by using the WLDM (red solid vertical line) and 300.5, 478.5, and 295.5 by using the GGDM (green dashed vertical line).

The essence of the Auto-Bayes method is that all potential emitters are fully analyzed, *i.e.*, for each locally brightest spot in an image the photoelectron count *n*_*psf*_ and localization is determined by PSF fitting. Figure 1a–c show the distribution of the determined photoelectron counts of all potential emitters in three training datasets from Sage *et al*.^7^. It is evident that the distributions of the false and true emitters are distinct from each other. As one would expect, the false emitters tend to have a lower photoelectron count. However, it is also clear the distribution of false emitters overlaps with that of the true emitters. Therefore, there exists no threshold that fully separates false and true emitters. To determine the optimal thresholds we analyzed the photoelectron count distributions with the generalized minimum error thresholding algorithm (GMET^18^), which is based on an unsupervised Bayesian method. The GMET algorithm requires *a priori* distribution models for both false and true emitters. We found that for the three training datasets, as well as many others, a reasonable threshold can be extracted using the following false-true distribution models: Gaussian-Gaussian (GGDM) and Weibull-Lognormal (WLDM)^19^,^20^. See Methods for implementation details. Notably, comparing the obtained thresholds using either the GGDM or WLDM shows that the GGDM generally results in a lower threshold. It is possible to determine quality metrics (Jaccard, Precision, Recall^7^) for the analysis of the training datasets using the evaluation tool CompareLocalization^21^ (see Table 1). For clarity, we also plotted the quality metrics as function of the threshold value (Fig. 1d–f). The thresholds found for both the GGDM and WLDM are consistently close to the optimal Jaccard index, where the GGDM tends to sacrifice certain precision for a higher recall. Note that the absolute value of the optimal Jaccard index is related to the analysis difficulty of the dataset^7^.

**Figure 2:**
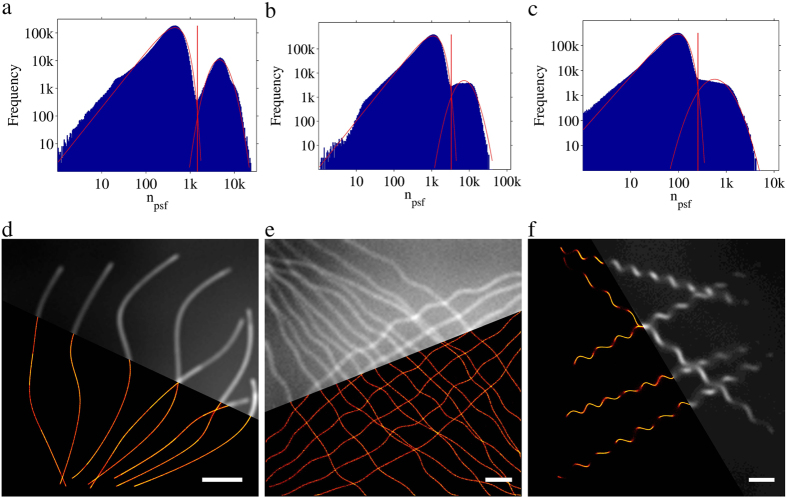
Auto-Bayes automatic threshold analyses for contest datasets^7^ from ISBI Challenge. (**a**–**c**) Distributions of n_psf_ (blue) with GMET analysis using the WLDM (red solid curves) for LS1, LS2 and LS3 datasets, respectively. Obtained *n*_*psf*_ thresholds are 1454.5, 3311.5, and 256.5, respectively (red solid vertical line). (**d**–**f**) Are cumulative (sum of all raw-image frames) and super-resolution images for LS1, LS2 and LS3 datasets, respectively. Scale bars are 2 μm.

We also analyzed the Long Sequence (LS) series of contest datasets from Sage *et al*.^7^. Like in real datasets, the positions of the emitters are unknown to us. A summary of the analysis of these datasets is shown in Fig. 2. The photoelectron count distribution of each contest dataset has a clear bimodal distribution (Fig. 2a–c), *i.e.*, there appears to be distinct distributions of false and true emitters that partially overlap. Here the estimated distributions for the false and true emitters using the WLDM are superposed on the distributions. The equivalent analysis using the GGDM is shown in Supplementary Fig. 2. The obtained thresholds for the LS1, LS2, and LS3 datasets for both distribution models are collected in Table 1. The datasets simulate tubulin structures. As expected, the tubulin structures in the reconstructed super-resolution images are much narrower than those in the cumulative fluorescence images obtained from summation of all single molecule images of the contest data sets (Fig. 2d–f). Comparison of the analysis with the WLDM and GGDM shows that the estimated distributions for the WLDM match better to the raw distribution than those obtained for the GGDM. The quality metrics that were determined to score the quality of the analysis of the competition datasets are collected in Table 1 together with the thresholds obtained using the WLDM and GGDM. The quality metrics are consistent with what we have seen with the training datasets, where the WLDM sacrifices recall for precision. In comparison with other software the Auto-Bayes method scores very well with regard to accurate single molecule detection (*i.e.*, high Jaccard)^7^.

**Figure 3:**
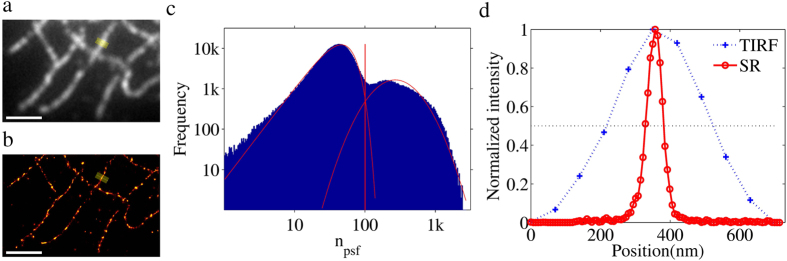
*d*STORM imaging of β-tubulin immunostaining in a HL-1 cell. (**a**) TIRF image. (**b**) Super-resolution images reconstructed by using Auto-Bayes with the WLDM from a sequence of 4000 single molecules images. (**c**) Distribution of *n*_*psf*_ (blue) with using a logarithmic scale and Auto-Bayes automated threshold analysis using the WLDM (red solid curves). The *n*_*psf*_ threshold (red solid vertical line) is 101.5 and 82959 single molecules were found. (**d**) Line profiles of a tubulin structure (marked by the yellow boxes in (**a**,**b**)); projection on the solid yellow line in (**a**,**b**). Scale bars are 2 μm.

To compare the analysis of simulated data with the analysis of real data, we performed *d*STORM^4^,^22^ measurements on microtubules in HL-1 cells. The microtubules were labelled using primary and secondary anti-bodies, where the latter was labelled with Alexa Fluor 647. A summary of the analysis is shown in Fig. 3. Note that the obtained photoelectron count distribution is bimodal and similar to the ones from the simulated datasets (Figs 1 and 2). The resulting reconstructed image shows the expected improvement in resolution over the conventional microscope image, as evidenced by the found microtubule width of ~50 nm for the *d*STORM measurement vs. ~300 nm for the conventional image (Fig. 3d). A similar result was obtained when the GGDM was used for the automatic threshold determination (see Supplementary Fig. 3). Again, the *n*_*psf*_ threshold obtained with the GGDM is slightly lower (by 4%; see Supplementary Table 1) compared to that obtained with the WLDM in accordance with the results on the simulated datasets (see Figs 1 and 2 and Supplementary Table 1). Consequently, the number of identified single molecule emitters is slightly but insignificantly higher (by 2.5%; Supplementary Table 1).

**Figure 4:**
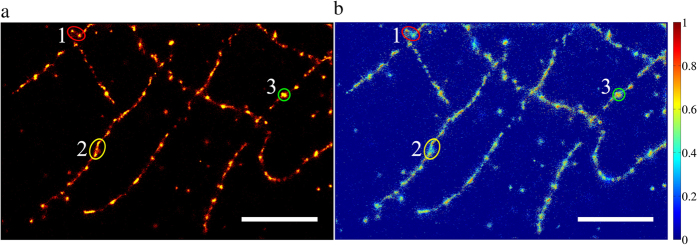
Super-resolution and reliability images for *d*STORM imaging of β-tubulin of a HL-1 cell. (**a**) Super-resolution and (**b**) reliability map for β-tubulin of a HL-1 cell. Dataset was analyzed by Auto-Bayes using the WLDM. Scale bars are 2 μm. Three pairs of numbered (and color coded) ovals indicate three regions of interest comparing the super-resolution image (**a**) with its reliability map (**b**).

The estimated distributions for both the false and true emitters can be used to determine a measure of confidence for each identified emitter. The probability of an emitter to be a true positive, in other words, the reliability of an emitter, can be defined as *R*(*n*_*psf*_) = *NT*(*n*_*psf*_)/[*NF*(*n*_*psf*_) + *NT*(*n*_*psf*_)]. Here *NT*(*n*_*psf*_) and *NF*(*n*_*psf*_) denote the number of true and false emitters respectively for a specific *n*_*psf*_, based on the estimated distributions. Accordingly, a reliability map of a reconstructed super-resolution image can be made with the normalized mean reliability of all potential emitters for each rendered pixel. The reliability map not only provides a simple way to judge the quality of the obtained super-resolution image. It may enable an advanced user to evaluate the trustworthiness of certain structures in a SMLM-image or to prompt improvements to the experimental design. As demonstrated in Fig. 4, while most of the tubulin structures in the SMLM image have a high reliability, there are also areas where the confidence is relatively low compared to the rest of the image. The potential usefulness of reliability maps falls into place comparing the different encircled areas of tubulin structures in Fig. 4a,b. Examples are highlighted in ovals 1, 2 and 3. Two dots with similar number of localizations are found in oval 1. The structure on the left was evidently more reliable than the neighboring point towards the right. The tubulin structure in oval 2 has a part with low density and low reliability connecting two regions with high reliabilities. According to the conventional wide-field fluorescence image (Fig. 3a), this tubulin structure has a high fluorescence intensity. The low reliability streak in the middle of the structure points towards non-ideal single molecule imaging conditions possibly due to a higher number of single molecules fluorescing at the same time that could result in miss-identification of single emitters. The area of the structure in oval 3 is larger compared to other dotted structures in the same image indicating that it may be composed by several sub-structures. Interestingly, this is also implied by the reliability map that shows this area is divided by zones with low reliabilities possibly reflecting the sub-structures. The equivalent analysis where the GGDM was used is shown in Supplementary Fig. 4.

**Figure 5:**
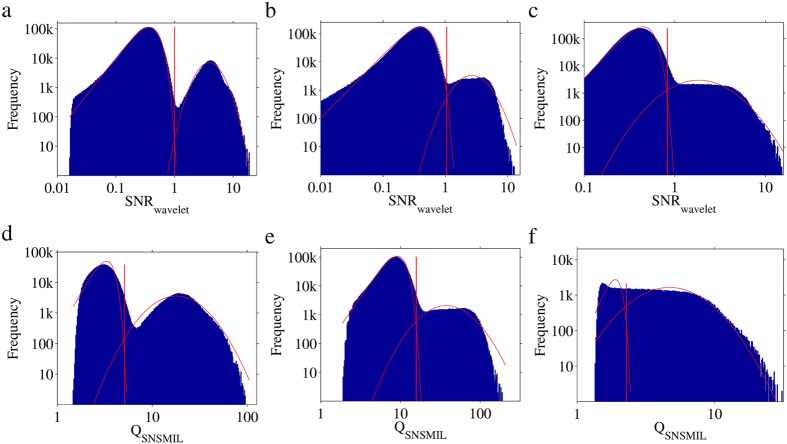
SNR_wavelet_ and Q_SNSMIL_ distributions for contest datasets^7^ from ISBI Challenge in 2015 analyzed by wavelet segmentation algorithm^9^,^10^ and SNSMIL^8^. (**a–c**) Distributions of SNR_wavelet_ (blue) and automated threshold analysis using the WLDM (red solid curves)for LS1, LS2 and LS3 datasets respectively. The obtained SNR_wavelet_ thresholds are 0.9976, 1.0509, and 0.8296 respectively (red solid vertical line). (**d–f**) Distributions of Q_SNSMIL_ (blue) and automated threshold analysis using the WLDM (red solid curves) for LS1, LS2 and LS3 datasets respectively. The obtained Q_SNSMIL_ thresholds are 5.1207, 15.7936, and 2.2808 respectively (red solid vertical line).

The principle of Auto-Bayes discussed above is not limited in its application to the photoelectron count of emitters. This principle can be adapted to the threshold determination of any other characteristic if the characteristic is distributed in a bimodal fashion with respect to false and true emitters. As an example, we applied the same principle to the selection characteristic SNR_wavelet_ used in the software package ThunderSTORM^10^, which makes use of a wavelet segmentation algorithm^9^,^10^ to filter the single molecule emitter images. The selection characteristic SNR_wavelet_ is defined as (Peak Intensity)/std(Wave. F1). We implemented the wavelet segmentation algorithm in MATLAB^®^ in order to determine the SNR_wavelet_ value for all emitter candidates (Supplementary Software 3). We find that the distribution of the SNR_wavelet_ parameter appears to have distinct distributions for false and true emitters for the LS1, LS2 and LS3 contest datasets (Fig. 5a–c). Analysis of the distributions with the GMET method using the WLDM (Fig. 5a–c) and GGDM (Supplementary Fig. 5a–c) shows that suitable thresholds can be determined. The obtained thresholds are listed in Supplementary Table 2.

SNSMIL^8^ is a SMLM method that makes use of a single user-dependent characteristic, Q_SNSMIL_. The threshold of Q_SNSMIL_ was determined using the Auto-Bayes method. We found that the distribution of the Q_SNSMIL_ parameter appears to have distinct distributions for false and true emitters for the LS1 and LS2 contest datasets (Fig. 5d,e), but that for the LS3 dataset, which has the worst image conditions, this distinction is less obvious (Fig. 5f). Thresholds determined by the WLDM (Fig. 5d–f) and GGDM (Supplementary Fig. 5d–f) are compared with those manually found by several users (see Supplementary Table 2). Here large deviations are especially found for the more difficult to analyze LS3 dataset, where the automatically determined threshold is sub-optimal in our opinion as the obtained false and true distributions are clearly sub-optimal (see Fig. 5f). It demonstrates that the automatic determination of a threshold using Auto-Bayes principle is only valid when true and false emitters are gathered in distinguishable groups with respect to the characteristic used for thresholding.

## Discussion

One of the major hurdles for making single molecule localization microscopy suitable for general use is the data analysis, which tends to be far from straightforward and can suffer from user bias. Automatic analysis protocols that take away this user bias are therefore highly desirable, not only for casual users, but also for expert users. Here we launch and explore a possibility to achieve fully automatic analysis of SMLM data. We introduce the Auto-Bayes method which makes use of information from both false and true emitters. By including the false emitters in the analysis, it is not only possible to determine the optimal threshold to use for discarding false emitters, but it is also possible to extract additional information for an emitter in the form of a reliability index. In contrast, the majority of analysis strategies mostly ignore information contained in potential emitters that are deemed either false emitters or to have a high probability of being false emitters.

Based on the training datasets kindly provided to the community by Sage *et al*.^7^, we were able to show that the Auto-Bayes method is able to find, based on the photoelectron count of an emitter, the optimal cutoff threshold that decides whether it is a false or true emitter. The method appears to consistently be able to find the threshold that leads to the optimal or close to optimal Jaccard index (see Fig. 1d–f), *i.e.*, the optimal balance between the found true-emitters and the number of false positives. Essentially, finding this optimal balance is something a user would do when manually optimizing an analysis. As such our goal to find an automatic analysis protocol has been achieved. Note that even though we used the photoelectron count of an emitter as a selection characteristic here, the Auto-Bayes method is not limited to using this particular characteristic. Any characteristic, that has distinct distributions with respect to false and true emitters, can be used. It is however important to determine whether these distinct distributions can be distinguished under SMLM imaging conditions. *E.g.*, we showed here that the selection characteristic SNR_wavelet_ obtained in the wavelet segmentation algorithm^9^,^10^ is suitable for the Auto-Bayes method. On the other hand, for the selection characteristic Q_SNSMIL_ used in SNSMIL^8^ there are cases where it is more difficult or maybe even impossible to confidently distinguish false and true emitters. In any case a suitable distribution model needs to be determined for each chosen characteristic. Here we have only used the WLDM and GGDM, which work adequately for the photoelectron count selection characteristic. GGDM was built based on central limit theorem and can be seen as a general choice. WLDM represents the photon intensity distribution of fluorescent emission by single molecule revealed by experimental measurement^20^. We found that WLDM sacrifices recall for precision by keeping only high reliable emitters. Other selection characteristics may require different distribution models to obtain adequate results. A tool coded in MATLAB^®^ is also provided for checking the validity visually (Supplementary Software 2). Note that the point of the used distributions is to find the best possible threshold level. It is not to describe the actual true-emitter distribution, as this would be unpractical. *E.g.*, from the data in Fig. 1a–c, it is clear that the true-emitter distribution consists of multiple sub-distributions that vary between datasets. Therefore the choice of the distribution model can be considered an implementation detail that a non-expert user should not have to worry about. It is for the experts to determine which distribution model works best with which characteristic. Essentially, the Auto-Bayes method uses *a priori* distributions that try to match the false and true emitter distributions to determine the optimal cutoff threshold. A shift in the threshold value will result in a shift in the balance between false positive and false negative emitters. Based on the determined false and true emitter distributions, a reliability index for an emitter was defined. It enables one to make an informed decision when interpreting specific super-resolved structures. Nevertheless, the quality of this reliability index is dependent on how well the used distribution model matches the false and true emitters. Correctly interpreting a reliability map, such as the one shown in Fig. 4b, not only helps with deciding which structures to trust or focus on, but it may also provide information about local, non-ideal measurement conditions in the sample. *E.g.*, a lower reliability may infer that the structure in question may be slightly outside the imaging plane when dealing with two-dimensional SMLM imaging or that locally too many single molecules were fluorescing at the same time.

In general, analysis of SMLM data is a multistep process. To determine potential emitters is a relatively fast process, for instance identifying the locally brightest pixels. Further analysis of these potential emitters tends to be computationally intensive. By discarding potential emitters with low probability of being true emitters, the time needed for analysis is significantly shortened. The tradeoff is that potentially useful information is discarded. The Auto-Bayes method makes use of information from both false and true emitters, since it does not try to distinguish or classify the locally brightest emitters immediately. Instead, it uses the distribution of the analysis characteristic (in Auto-Bayes, the photoelectron count) to determine the optimal threshold for separation of false and true emitters automatically, given that the distributions for false and true-emitters are distinguishable. The renunciation of a pre-evaluation of possible emitters before single emitter localization comes at the cost of enhanced computational effort. By fully analyzing all candidate emitters, the number of emitters that need to be analyzed can increase by a factor of 10 to 100 (see Supplementary Table 1). This is clearly illustrated by the distributions of the photoelectron count of emitters shown in Figs 1a–c, 2a–c and 3c, where the implied subset of true emitters is always much smaller than the subset of false-emitters. The use of the parallel processing capabilities of modern graphic processing units (GPU), can however keep the computation times reasonable. In the light of fast development of computer hardware, real time analysis can be achieved possibly in the near future. There is a great hope that Auto-Bayes can be used in a real time manner that is to say, obtain the results immediately following the measurements.

Our implementation of the Auto-Bayes method that just makes use of the photoelectron count scored very well in a resent comparison with most of current analysis tools^7^. Especially with respect to the Jaccard index which the Auto-Bayes method is designed to excel at. Most importantly this was achieved in the absence of a human bias, as the result was obtained fully automatically, requiring only parameters related to experimental conditions, such as camera, optical parameters, and emission wavelength, to be provided for analysis. This first implementation can be considered as a proof of concept that has ample room for improvement. To start, only a basic emitter analysis algorithm was used. Advancements here should make it possible to improve the localization accuracy of emitters. Furthermore, we only used a single selection characteristic. Using multiple selection characteristics will potentially enable a better separation of false and true emitters. *E.g.*, based on one characteristic two emitters may fall into the overlap of the distribution of false and true emitters while for another characteristic they may be separated. In addition it may be possible to distinguish additional subsets in the true-emitter subset when multiple selection characteristics are used. Detection of multiple overlapping emitters also can be implemented when using an appropriate multiple PSFs fitting. Such an algorithm might even be easier to implement as the algorithm has to take less care with respect to generating false positives, as these should be filtered out using the automatic thresholding algorithm afterwards. Alternatively, the method may be applied in other areas in analysis strategies were thresholds are used for parameters that have distinguishable distributions of false and true emitters. *E.g.*, if a strategy relies on a threshold in pre-selection of potential emitters it could be used there as well. In the end it is a question of costs and benefits on where and how automatic threshold determination is used.

Recently Smith *et al*.^23^ suggested an alternative method for user-intervention free single molecule localization using a generalized likelihood ratio test that allows to minimize the number of false negatives at a fixed number of false positives. Unlike the Auto-Bayes method that we present here, Smith *et al*. describe a more specific method that cannot easily be used to complement existing analysis methods. However, combining the strengths of both methods may result in higher quality analysis that neither method could reach on their own.

In conclusion, we have shown a proof of concept for a fully automated analysis method for use with SMLM data. Though we have mainly demonstrated the use of this concept, the Auto-Bayes method, using the photoelectron count of emitters as selection characteristic, the method should be applicable using others as well. As such it should be fairly straightforward to incorporate it in other analysis tools, allowing those tools to also provide the highly sought after fully automated analysis capability. As experience in the use of this method grows, more sophisticated automatic analysis schemes may become reality in the near future.

## Methods

### Auto-Bayes implementation

The source code and binaries of the implementation of the Auto-Bayes method we used is available as Supplementary Software 1 (named Auto-Bayes). See Supplementary Note 2 for license and hardware requirement details. The Auto-Bayes method was implemented such that each image in the SMLM dataset is analyzed separately in a 5-step algorithm. The combined results are then analyzed further using Bayesian statistics to determine the optimal emitter photoelectron count threshold. See Supplementary Fig. 1 for a flow chart describing image processing by the Auto-Bayes software.

#### Image Analysis Step 1: Source-image conversion

The pixel values of the source image are converted to photoelectron counts using Equation 1^8^. Here *n*(*i*, *j*) is the photoelectron count of pixel (*i*, *j*); *I*(*i*, *j*) is the value of pixel (*i*, *j*); *I*_*offset*_ denotes the bias offset; *G*_*eff*_ is the applied effective electronic gain in AD counts per photoelectron (note for an EMCCD camera this value is dependent on the applied electron multiplication gain).


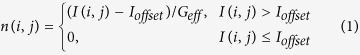


All subsequent image analysis steps are performed on the converted image data.

#### Image Analysis Step 2: Search for locally brightest spots

Image regions containing a locally brightest spot are defined by pixels that have the maximum photoelectron count value within an Airy disk radius of the pixel. The position of locally brightest pixel is denoted as (*i*_*peak*_, *j*_*peak*_). The radius of the Airy disc in the image plane is defined by Equation 2^8^. Here *R*_*airy*_ is the airy disk radius in pixels; *M* denotes magnification; *λ* is the wavelength of maximum fluorescence emission in nm; *μ* is the physical pixel size of the camera in nm per pixel; *NA* denotes the numerical aperture of the imaging system (usually defined by the used objective).


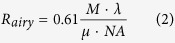


#### Image Analysis Step 3: Background image estimation

For use in step 4, the background signal for each pixel in the image needs to be estimated. The background image is estimated based on the Gaussian-smoothed image. The used Gaussian-filter is defined by Equation 3. Here *s*_0_ is the theoretical Gaussian width of the PSF as described by Equation 4^8^,^11^,^24^; and *h*_*f*_ is defined as the nearest greater or equal integer of the airy disk radius as defined by Equation 2 (*i.e.*, *ceil*(*R*_*airy*_)).


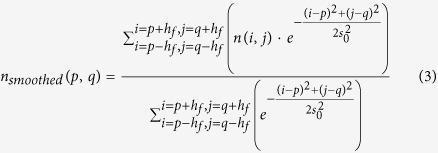



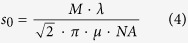


The estimated background signal of a pixel is the local minimum in the smoothed-image in a square region of size (6*h*_*f*_ + 1) centered on that pixel.

#### Image Analysis Step 4: Gaussian PSF fitting

The perfectly focused point spread function (PSF) model can be approximated as a shape fixed 2D Gaussian function as described by Equation 5^2^. Here *n*_*psf*_ denotes the photoelectron count of the PSF; *x*_0_ and *y*_0_ are the central positions in the *x* or *y* direction respectively in pixels; *n*_*b*_ is the local background; *s*_0_ is the theoretical Gaussian width of the PSF as described by Equation 4^11^,^24^.


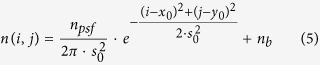


We use nonlinear regression to fit Equation 5 to the image regions containing the locally brightest spots determined in image analysis step 2. As such we need to determine reasonable starting values for the parameters, *x*_0_, *y*_0_, *n*_*b*_, and *n*_*psf*_ for each image region to be processed. For *x*_0_ and *y*_0_ the position of the locally brightest pixel *i*_*peak*_ and *j*_*peak*_ is chosen respectively. The initial value for *n*_*b*_ is chosen to be the value of pixel (*i*_*peak*_, *j*_*peak*_) in the background image generated in image analysis step 3. The initial value for *n*_*psf*_ is defined by Equation 6.


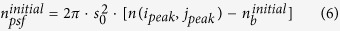


Nonlinear regression is performed on the data from the image generated in image analysis step 1. The actual region that is used for the nonlinear regression is the square region of size (2*h*_*f*_ + 1) centered on the coordinates of the locally brightest spot (*i*_*peak*_, *j*_*peak*_) of the image. The Levenberg-Marquardt algorithm implemented for GPU parallel computation^8^,^25^, is used to fit Equation 5 to the indicated fit region.

#### Image Analysis Step 5: Discard too close spots

If more than one brightest spot is found within a region with the radius of *s*_*0*_ as defined in Equation 4, only the spot with the largest photoelectron count is retained for further consideration.

#### Automatic threshold determination for emitter selection

A histogram, *h*(*n*_*psf*_), is generated for the emitter photoelectron count (*n*_*psf*_) based on the image analysis results of all images in the SMLM dataset. Here, *n*_*psf*_ is quantized into *N* gray levels {0, 1, …, *N−*1}, where *N* will typically be the nearest greater or equal integer of the maximum observed photoelectron count, but will never be greater than the number of retained brightest spots.

The obtained histogram is analyzed using a generalized minimum error thresholding algorithm (GMET)^18^. GMET is an unsupervised Bayesian thresholding method based on histogram analysis aimed to minimize the classification error. Equation 7 represents the generalized criterion function^18^, which when minimized yields the optimal threshold. Here 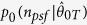 and 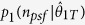 respectively represent the false and true emitter distribution, where 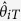 is the parameter vector of distribution *i*. Combined they form a distribution model. *P*_*iT*_ is defined by Equation 8, where *R*_*0T*_ denotes the set of false emitters ({0, 1, … *T*}) and *R*_*1T*_ denotes the set of true emitters ({*T*+1, *T*+2, …, *N−*1}).


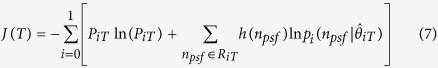



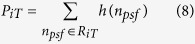


Auto-Bayes implements two *a priori* distribution models. The obtained optimal threshold is more precise when the distributions are more consistent with experimental datasets. The two *a priori* distribution models available in Auto-Bayes are Gaussian-Gaussian (GGDM) and Weibull-Lognormal (WLDM). For the GGDM both the false and true emitters are assumed to be Gaussian distributed, as implied by the central limit theorem. Equation 9 describes the Gaussian distribution, where *μ*_*i*_ and *σ*_*i*_ represent the distribution model parameters for Gaussian distribution *i*.


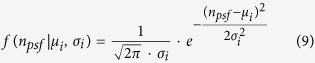


For the WLDM, the false emitters are assumed to follow the Weibull distribution^19^. Equation 10 describes the Weibull distribution, where *λ* and *k* represent the distribution model parameters.


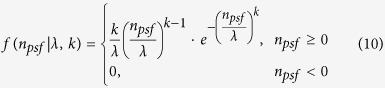


The true emitters are assumed to follow a lognormal distribution^19^,^20^,^26^. Equation 11 describes the lognormal distribution, where *μ* and *σ* represent the distribution model parameters.


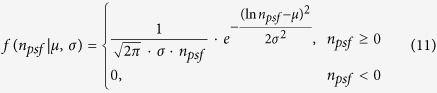


Of all processed emitters, the emitters with *n*_*psf*_ greater or equal to the threshold determined by Auto-Bayes software are exported. These exported localizations are subsequently used to reconstruct a super-resolution image. Also photoelectron information of all analyzed potential emitters, together with the distribution model parameters are subsequently exported as tabulated data. This table can be used to generate a photoelectron count histogram for either visualization (*e.g.*, using Supplementary Software 2) or custom analysis. If localizations below the automatically determined threshold needed to be exported as well, *e.g.*, when a reliability map needed to be generated, a manual threshold of 1 was set.

### Determination of quality metrics

We used four indexes Jaccard, Precision, Recall, and RMSD (root mean square distance) to evaluate the quality of the performed analyses as also used by Sage *et al*. to benchmark different software packages^7^. These indexes are defined by equations (12)–(15), 16, 17, 18. We denominate A and B as the simulated dataset (reference dataset) and the reconstructed dataset, respectively. Three relevant quantities are defined as true positive (*TP*), false positive (*FP*) and false negative (*FN*) as follows: the number of true emitters reconstructed, *TP* = *A* ∩ *B*; the number of false emitters reconstructed, *FP* = *B* − *TP*; and the number of true emitters missed, *FN* = *A* − *TP*.


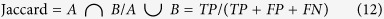



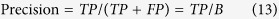



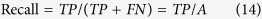



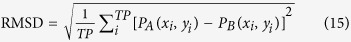


### Cell culture and immunostaining

The murine atrial cardiomyocyte cell line, HL-1, was kindly provided by Dr. W. C. Claycomb (Louisiana State University Health Sciences Center). Cell culture was performed as specified for the cell line^27^. Medium contained: fetal bovine serum (FBS) 10%, norepinephrine 0.1 mM, L-glutamine 2 mM and penicillin/streptomycin 100 U/ml in Claycomb medium. Exchange of media was done every 24–48 h. Before immunostaining, cells were split onto fibronectin pre-coated dishes after showing the macroscopic beating phenotype^27^. There are several steps for β-tubulin immunostaining in HL-1 cells. Cells were treated as follows. Step 1: washed 3 times with phosphate buffered saline (PBS), and then incubated in 4% paraformaldehyde (PFA) in PBS for 15 minutes. Step 2: incubated with 0.5% Triton X-100 for 10 minutes to increase the penetration of antibodies through cell membranes. Step 3: washed 3 times with 200 μl PBS for 5 minutes. Step 4: blocked with freshly made blocking buffer containing 5% normal goat serum (Sigma; G9023) in PBS for 45 minutes. Step 5: incubated with first antibody solution anti-β-tubulin (1 μl anti-β-tubulin solution: 500 μl blocking buffer, Invitrogen; 32–2600) for 60 minutes (antibody diluted in blocking buffer). Step 6: washed 6 times with 200 μl PBS supplemented with 0.1% Tween-20 for 5 minutes. Step 7: incubated with the second antibody solution anti-mouse (1 μl anti-mouse solution: 10000 μl blocking buffer, Invitrogen; A-21237) for 60 minutes (antibody diluted in blocking buffer) in the dark. Step 8: washed 6 times with 200 μl PBS supplemented with 0.1% Tween-20 for 5 minutes. Step 9: post-fixed with 4% PFA in PBS for 5 minutes. Step 10: washed 2–3 times with PBS for 5 minutes, then washed with PBS + NaN_3_ (0.1%) 1 time in the last round. Step 11: store at 4 °C.

### Wide-field/TIRF fluorescence microscope

A detailed description of the used microscope setup is presented elsewhere^8^. In short, the setup is based on an Olympus IX-71 inverted microscope body (Olympus, Hamburg, Germany). We made use of a 642 nm diode laser (LBX-642-130 CIR-PP; Oxxius, Lannion, France) to excite the sample in TIRF mode. All measurements were done with an ApoN 60x Oil TIRF objective (NA 1.49; Olympus) and a post-magnification resulting in an effective pixel-size of 70 nm/pixel. Excitation and emission light were separated via a multiband dichroic mirror (F73-866, BS R405/488/561/633; AHF Analysentechnik, Tübingen, Germany) in combination with a multiple bandpass filter (F72-866, 466/523/600/677; AHF Analysentechnik). Images were recorded with an EMCCD camera (Andor iXon DU897E; Andor, Belfast, UK) cooled to −75 °C.

### *d*STORM imaging

For the *d*STORM measurements the sample was excited with a 642 nm diode laser (100 mW output power). A series of 4000 TIRF images at a resolution of 70 nm/pixel was recorded for a single sample position. Images were recorded using an exposure time of 20 ms. The used imaging buffer contained a glucose oxidase based oxygen scavenger in combination with 90 mM β-mercaptoethylamine (MEA; Sigma-Aldrich, Taufkirchen, Germany).

## Additional Information

**How to cite this article**: Tang, Y. *et al*. Automatic Bayesian single molecule identification for localization microscopy. *Sci. Rep.* **6**, 33521; doi: 10.1038/srep33521 (2016).

## Supplementary Material

Supplementary Information

Supplementary Software 1

Supplementary Software 2

Supplementary Software 3

## Figures and Tables

**Table 1:** The performance of Auto-Bayes on training and contest datasets^7^ from ISBI Challenge in 2015.

Dataset	Threshold		Jaccard		Precision	
	GGDM	WLDM	GGDM	WLDM	GGDM	WLDM
Tubulins I	300.5	356.5	0.720	0.759	0.884	0.988
Tubulins II	478.5	556.5	0.702	0.677	0.956	0.997
Bundle of Tubulins	295.5	424.5	0.924	0.925	0.998	1.000
LS1	1107.5	1454.5	0.865	0.871	0.980	1.000
LS2	2759.5	3311.5	0.711	0.679	0.950	9.990
LS3	228.5	256.5	0.490	0.462	0.920	0.950
**Dataset**	**Recall**		**RMSD (nm)**		**Time (mm:ss)**	
	**GGDM**	**WLDM**	**GGDM**	**WLDM**	**GGDM**	**WLDM**
Tubulins I	0.796	0.766	29.166	28.431	6:50	6:57
Tubulins II	0.726	0.678	33.767	32.729	7:01	7:32
Bundle of Tubulins	0.926	0.925	22.278	22.220	5:49	6:02
LS1	0.877	0.872	28.270	28.030	10:31	10:41
LS2	0.737	0.683	44.410	41.430	16:51	17:15
LS3	0.512	0.473	63.640	59.840	16:18	16:19

Auto-Bayes was running on a Graphic Processing Unit (GPU) based computer (GPU: Nvidia GTX580) with 64 bit windows 7 operating system. The quality metrics (see Methods) were determined with the help of the evaluation tool CompareLocalization^21^. The tolerance radii were set as full width at half maximum (FWHM) = 258.21 nm for all three training datasets Tubulins I, Tubulins II and Bundle of Tubulins; the tolerance radii were set as FWHM = 224.32 nm, 258.21 nm, 247.60 nm for three contest datasets LS1, LS2 and LS3 respectively^7^.
