# Supplementary material for: Automatic Bayesian single molecule identification for localization microscopy
Source: Sci Rep. 2016 Sep 19;6:33521. doi: 10.1038/srep33521 (PMC5027599; doi:10.1038/srep33521)
Supplement: Supplementary Information [file srep33521-s1.pdf]

**Supplementary information**

# Automatic Bayesian single molecule identification for localization microscopy

Yunqing Tang[1], Johnny Hendriks[2], Thomas Gensch[2], Luru Dai[1,*] & Junbai Li[1,3,*]

[1] *National Center for Nanoscience and Technology of China, Beijing 100190, P.R. China.*
[2] *Institute of Complex Systems (ICS-4, Cellular Biophysics), Forschungszentrum Jülich, Jülich 52428, Germany.*
[3] *Institute of Chemistry, Chinese Academy of Sciences, Beijing 100190, P.R. China.*
[*] *To whom correspondence should be addressed*



| | |
|---|---|
| **Supplementary Figure 1** | Schematic illustration of Auto-Bayes algorithm. |
| **Supplementary Figure 2** | Auto-Bayes automatic threshold analyses for contest datasets with GGDM. |
| **Supplementary Figure 3** | Auto-Bayes automatic threshold analyses for tubulins of HL-1 cells with GGDM. |
| **Supplementary Figure 4** | Reliability analyses with Gaussian-Gaussian distribution model. |
| **Supplementary Figure 5** | Automatic threshold analyses of ThunderSTORM and SNSMIL for contest datasets with GGDM. |
| **Supplementary Table 1** | Thresholds and numbers of emitters detected by the GGDM and the WLDM. |
| **Supplementary Table 2** | Comparison of automatic and manual threshold analyses of ThunderSTORM and SNSMIL for contest datasets. |
| **Supplementary Note 1** | Parameters settings. |
| **Supplementary Note 2** | Observations on usage of Auto-Bayes. |

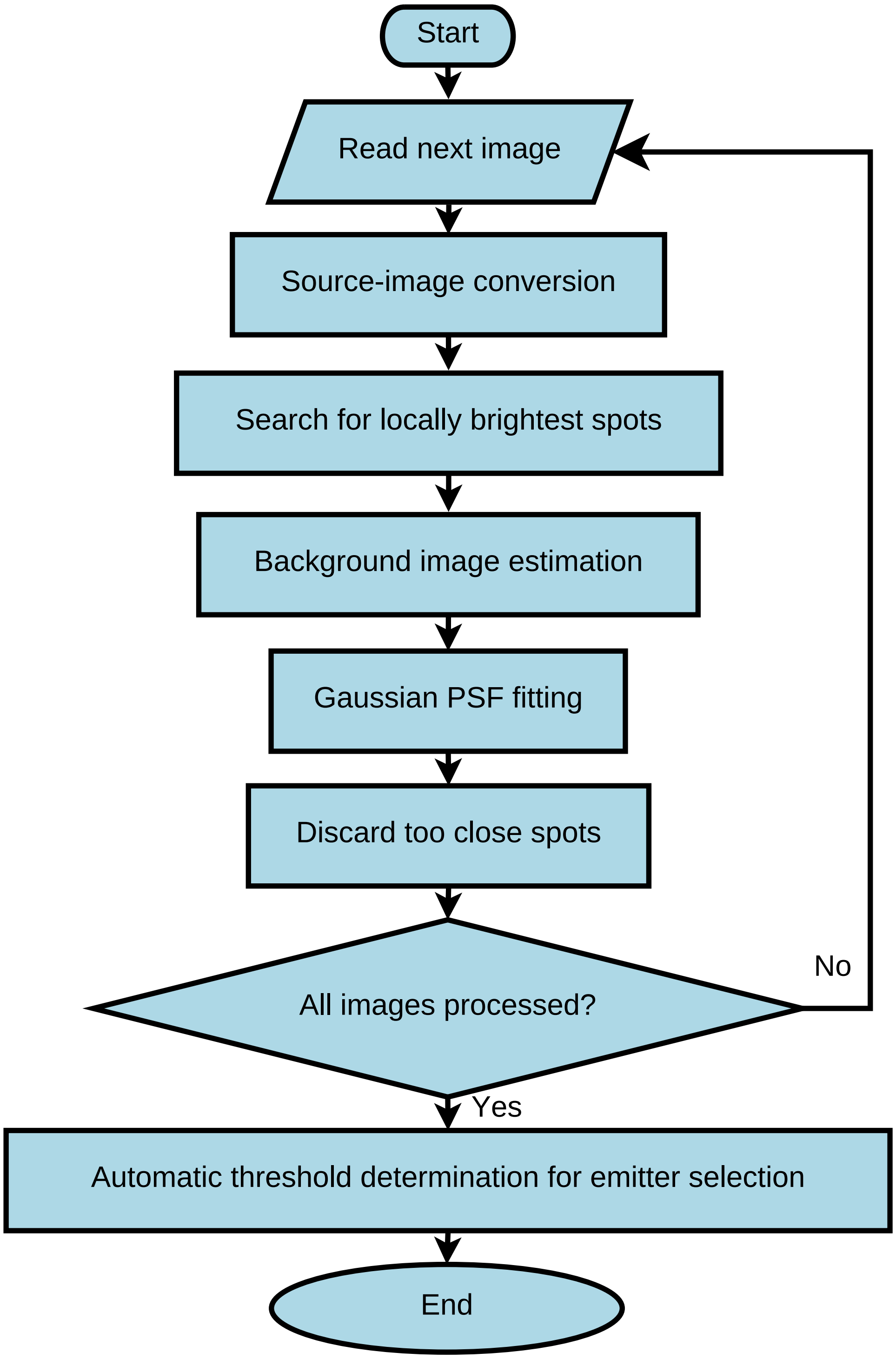


**Supplementary Figure 1** Schematic illustration of the Auto-Bayes algorithm. For a detailed description of algorithm, see **Methods**.

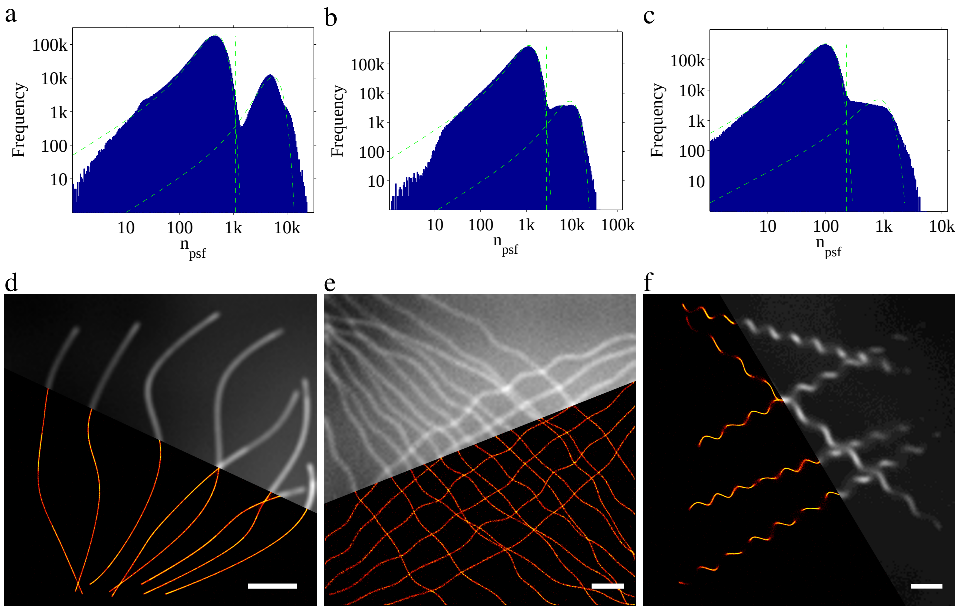


**Supplementary Figure 2** Auto-Bayes automatic threshold analyses for contest datasets [1] from ISBI Challenge in 2015. (a), (b) and (c) Distributions of $n_{psf}$ (blue) using a logarithmic scale and Auto-Bayes automated threshold analyses with GGDM (green dashed curves) for LS1, LS2 and LS3 datasets respectively, and the $n_{psf}$ thresholds are 1107.5, 2759.5, 228.5 respectively (green dashed vertical line). (d), (e) and (f) are cumulative (sum of all raw-image frames) and super-resolution images for LS1, LS2 and LS3 datasets respectively. Scale bars are 2 µm.

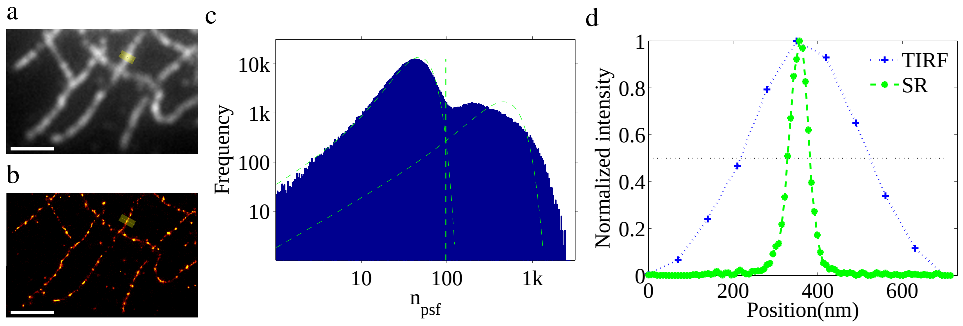


**Supplementary Figure 3** *d*STORM imaging of $\beta$-tubulin immunostaining in a HL-1 cell. (a) TIRF image. (b) Super-resolution images reconstructed by using Auto-Bayes with GGDM from a sequence of 4000 single molecules images. (c) Distribution of $n_{psf}$ (blue) using a logarithmic scale and Auto-Bayes automated threshold analysis with GGDM (green dashed curves). The $n_{psf}$ threshold (green dashed vertical line) is 97.5 for GGDM (green dashed vertical line) and 85092 single molecules are found. (d) Line profiles of a tubulin structure (marked by a yellow box in (a) and (b)). Scale bars are 2 µm.

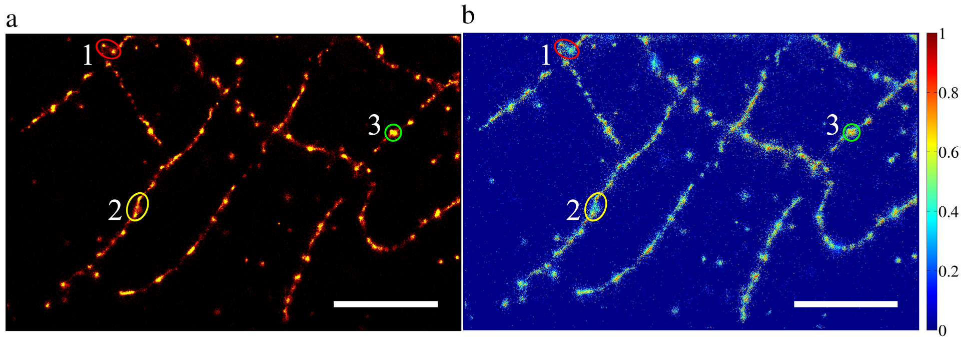


**Supplementary Figure 4** Super-resolution and reliability images for $\beta$-tubulin of a HL-1 cell. (a) super-resolution and (b) its reliability map for $\beta$-tubulin of a HL-1 cell. Dataset was analyzed by Auto-Bayes with GGDM. Scale bars are 2 µm. Three pairs of colored circles indicate three regions of interest comparing super-resolution image and its reliability map.

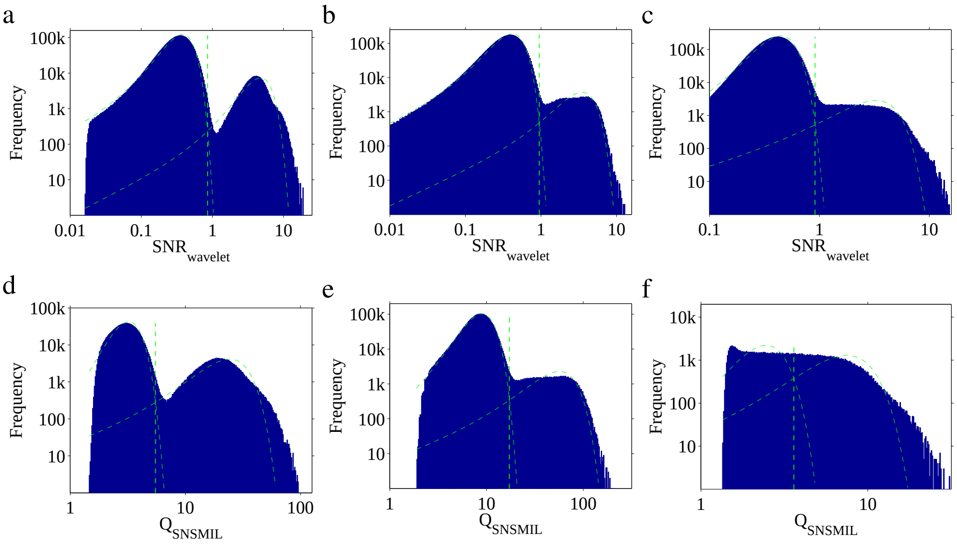


**Supplementary Figure 5** $\mathrm{SNR}_{\mathrm{wavelet}}$ and $\mathrm{Q}_{\mathrm{SNSMIL}}$ distributions for contest datasets [1] from ISBI Challenge in 2015 calculated by wavelet segmentation algorithm [2, 3] and SNSMIL [4]. (a), (b) and (c) Distributions of $\mathrm{SNR}_{\mathrm{wavelet}}$ (blue) and automated threshold analysis with GGDM (green dashed curves) using a logarithmic scale for LS1, LS2 and LS3 datasets respectively, the $\mathrm{SNR}_{\mathrm{wavelet}}$ thresholds are 0.8566, 0.9491, 0.9134 respectively (green dashed vertical line). (d), (e) and (f) Distributions of $\mathrm{Q}_{\mathrm{SNSMIL}}$ (blue) and automated threshold analysis with GGDM (green dashed curves) using a logarithmic scale for LS1, LS2 and LS3 datasets respectively, the $\mathrm{Q}_{\mathrm{SNSMIL}}$ thresholds are 5.4984, 17.2013, 3.6041 respectively (green dashed vertical line).

**Supplementary Table 1** Thresholds and numbers of emitters detected by the GGDM and the WLDM. Auto-Bayes was applied on long sequence training and contest datasets from ISBI Challenge in 2015 [1] and *d*STORM imaging data.

| Datasets | Num. of potential emitters | Model | $n_{psf}$ threshold | Num. of true emitters |
|---|---|---|---|---|
| Tubulins I | 9,118,056 | GGDM | 300.5 | 90,033 (1.0%) |
| | | WLDM | 356.5 | 77,459 (0.8%) |
| Tubulins II | 9,449,238 | GGDM | 478.5 | 75,948 (0.8%) |
| | | WLDM | 556.5 | 68,072 (0.7%) |
| Bundle of Tubulins | 1,154,135 | GGDM | 295.5 | 75,175 (6.5%) |
| | | WLDM | 424.5 | 74,939 (6.5%) |
| LS1 | 5,620,270 | GGDM | 1107.5 | 258,043 (4.6%) |
| | | WLDM | 1454.5 | 252,560 (4.5%) |
| LS2 | 11,756,957 | GGDM | 2759.5 | 145,045 (1.2%) |
| | | WLDM | 3311.5 | 129,728 (1.1%) |
| LS3 | 10,894,657 | GGDM | 228.5 | 194,113 (1.8%) |
| | | WLDM | 256.5 | 175,390 (1.6%) |
| $\beta$-Tubulin imaging | 556,050 | GGDM | 97.5 | 85,092 (15.3%) |
| | | WLDM | 101.5 | 82,959 (14.9%) |

**Supplementary Table 2** Comparison over automated thresholding by the WLDM, the GGDM and manual thresholding of $\mathrm{SNR}_{\mathrm{wavelet}}$ from wavelet segmentation algorithm [2, 3] and $\mathrm{Q}_{\mathrm{SNSMIL}}$ from SNSMIL [4] on long sequence contest datasets from ISBI Challenge in 2015 [1].

| Characteristics | Datasets | WLDM | GGDM | Manual |
|---|---|---|---|---|
| $\mathrm{SNR}_{\mathrm{wavelet}}$ | LS1 | 0.9976 | 0.8566 | 0.9875±0.0854 |
| | LS2 | 1.0509 | 0.9491 | 1.1125±0.0853 |
| | LS3 | 0.8296 | 0.9134 | 0.9750±0.0646 |
| $\mathrm{Q}_{\mathrm{SNSMIL}}$ | LS1 | 5.1207 | 5.4984 | 6.200±0.4320 |
| | LS2 | 15.7936 | 17.2013 | 14.8750±0.6292 |
| | LS3 | 2.2808 | 3.6041 | 1.8250±0.3403 |

# 1 Supplementary Note 1

All input parameters are hardware-related. For long sequence Tubulins I training dataset, those parameters set as follow, numerical aperture: 1.4, optical magnification: 1, emission wavelength (nm): 723, gain: 1, $e^-$/AD count: 1, pixel diameter (nm): 150, bias offset (AD counts): 0.

For long sequence Tubulins II training dataset, those parameters set as follow, numerical aperture: 1.4, optical magnification: 1, emission wavelength (nm): 723, gain: 1, $e^-$/AD count: 1, pixel diameter (nm): 150, bias offset (AD counts): 0.

For long sequence bundled of Tubulins training dataset, those parameters set as follow, numerical aperture: 1.4, optical magnification: 1, emission wavelength (nm): 723, gain: 1, $e^-$/AD count: 1, pixel diameter (nm): 100, bias offset (AD counts): 0.

For LS1 contest dataset, those parameters set as follow, numerical aperture: 1.46, optical magnification: 1, emission wavelength (nm): 655, gain: 1, $e^-$/AD count: 1, pixel diameter (nm): 100, bias offset (AD counts): 0.

For LS2 contest dataset, those parameters set as follow, numerical aperture: 1.4, optical magnification: 1, emission wavelength (nm): 723, gain: 1, $e^-$/AD count: 2, pixel diameter (nm): 150, bias offset (AD counts): 0.

For LS3 contest dataset, those parameters set as follow, numerical aperture: 1.46, optical magnification: 1, emission wavelength (nm): 723, gain: 1, $e^-$/AD count: 1, pixel diameter (nm): 100, bias offset (AD counts): 0.

For experimental tubulins dataset, those parameters set as follow, numerical aperture: 1.49, optical magnification: 228.57, emission wavelength (nm): 668, gain: 300, $e^-$/AD count: 11.9, pixel diameter (nm): 16000, bias offset (AD counts): 100.

# 2 Supplementary Note 2

We provide three pieces of Supplementary Software. **Supplementary Software 1** contains the Auto-Bayes software with user-guide and is released under BSD and LGPL licenses. It is free for both academic and commercial use. Auto-Bayes needs a computer equipped with a NVidia CUDA-enabled graphics card that has compute capability 2.0 or higher. The latest version of the Auto-Bayes software can be found at http://english.nanoctr.cas.cn/dai/software/.

**Supplementary Software 2** is a histogram visualization tool for output generated by **Supplementary Software 1**. **Supplementary Software 3** implements the wavelet segmentation algorithm, which is also used by ThunderSTORM [3], to detect emitters from SMLM data. Our implementation was designed to determine if the Auto-Bayes method can be applied to automatically determine the optimal $\mathrm{SNR}_{\mathrm{wavelet}}$ threshold value for this algo-

rithm. Both **Supplementary Software 2** and **3** were coded in MATLAB®, and have a BSD license.

## References


[1] Sage, D. *et al.* Quantitative Evaluation of Software Packages for Single-Molecule Localization Microscopy. *Nat. Methods* **12**, 717-724 (2015).

[2] Izeddin, I. *et al.* Wavelet analysis for single molecule localization microscopy. *Opt. Express* **20**, 2081-2095 (2012).

[3] Ovesný, M., Křížek, P., Borkovec, J., Švindrych, Z. & Hagen, G. M. ThunderSTORM: a comprehensive ImageJ plugin for PALM and STORM data analysis and super-resolution imaging. *Bioinformatics* **30**, 2389-2390 (2014).

[4] Tang, Y. *et al.* SNSMIL, a real-time single molecule identification and localization algorithm for super-resolution fluorescence microscopy. *Sci. Rep.* **5**, 11073 (2015).
