# Supplementary figures and images for: Automatic Bayesian single molecule identification for localization microscopy
Source: Sci Rep. 2016 Sep 19;6:33521. doi: 10.1038/srep33521 (PMC5027599; doi:10.1038/srep33521)

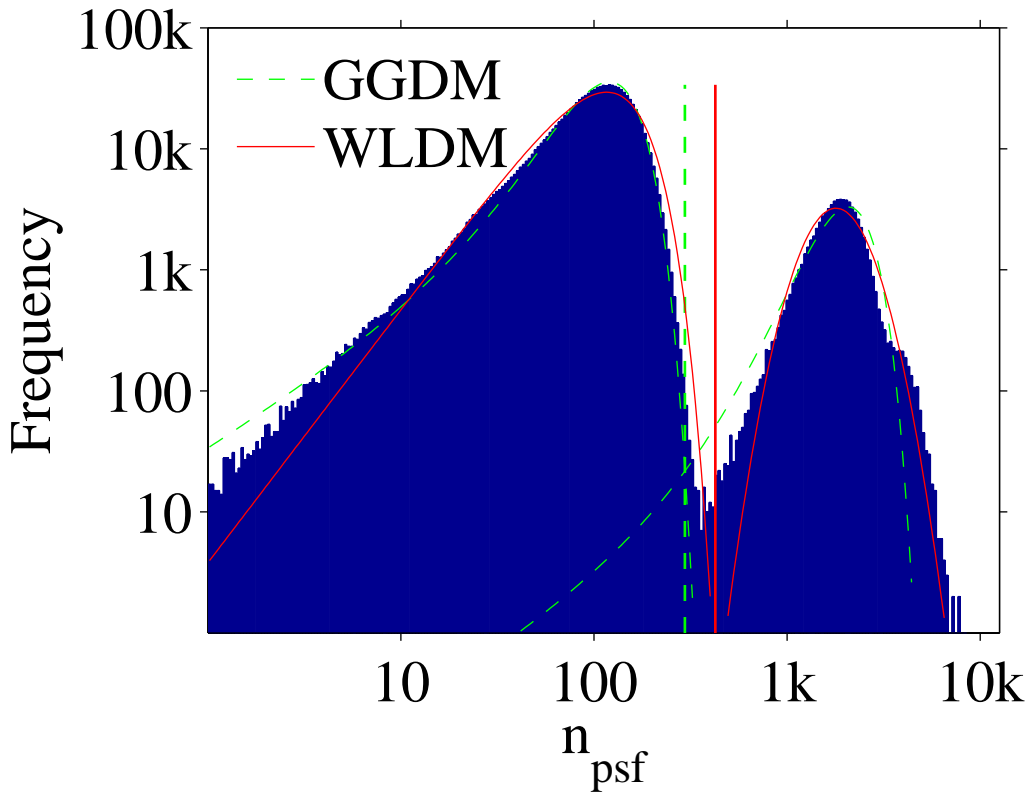

Supplement: Supplementary Software 2 [file srep33521-s3.zip › SupplementarySoftware2/BundledofTubulins_histogram_GG_WL.pdf]
