# Supplementary material for: Automatic Bayesian single molecule identification for localization microscopy
Source: Sci Rep. 2016 Sep 19;6:33521. doi: 10.1038/srep33521 (PMC5027599; doi:10.1038/srep33521)
Supplement: Supplementary Software 1 [file srep33521-s2.zip › SupplementarySoftware1/AutoBayes_linux_v1.0.zip!/AutoBayes_linux_v1.0/user's guide.pdf]

Auto-Bayes User's Guide

Version 1.0, date: 2015-02-09

Yunqing Tang tangyunqing@cqu.edu.cn *& Luru Dai* dai@nanoctr.cn

Auto-Bayes is a software package based on Bayesian statistics and requires no user dependent parameters for molecule detection and image reconstruction of single-molecule localization microscopy (SMLM), including photoactivated localization microscope (PALM), stochastic optical reconstruction microscope (STORM), and direct stochastic optical reconstruction microscopy (dSTORM), *etc*. Based on the distribution of photoelectrons for all candidates of single emitters, Bayesian statistics is used with two prior distributions for false and true emitters respectively, Auto-Bayes automatically discriminate single molecules with high accuracy. It is a cross-platform parallel-computing software package running on Graphic Processing Unit (GPU) for 2-dimensions and 3-dimensions (under development) SMLM data analysis.

The motivation of the development of Auto-Bayes is to offer a SMLM software package without requiring any user dependent parameters, and give high-accuracy detection of single molecule (High Jaccard). It is useful for the researchers using SMLM without being experts in single molecule spectroscopy and super-resolution fluorescence microscopy.

Auto-Bayes depends on libtiff, LGPL v2 Qt 4.8.4 dynamic library and CUDA runtime dynamic library. Auto-Bayes is released under a BSD and LGPL license. Auto-Bayes runs on a computer equipped with Nvidia CUDA-enabled graphics card with compute capability 2.0 or higher. To determine compute capability of an Nvidia graphics card, please refer http://developer.nvidia.com/cuda/cuda-gpus.

In Auto-Bayes, all inputs are "hardware" related and picture rendering parameters as shown in see **Fig 1**. Input parameters including: numerical aperture of objective, optical magnification, emission wavelength (unit: nm, the wavelength of maximum fluorescence emission), camera gain, e-/AD count (depends on camera performance and settings, see **Fig 3** for a case of Andor EMCCD), camera pixel diameter (unit: nm, in detector plane), camera bias offset (in the unit of AD counts that is 100 for Andor DU897 EMCCD). Rendering options including magnification, rendering color map. PSF fitting is done for all candidates (local maxima in the image). A threshold is defined to classify emitters from fake candidates. The threshold can be defined automatically by Bayesian statistics with Weibull-Lognormal distribution (recommended) and Gaussian-Gaussian distribution. A user selected threshold also can be used for trial and examination, see **Fig 2**.

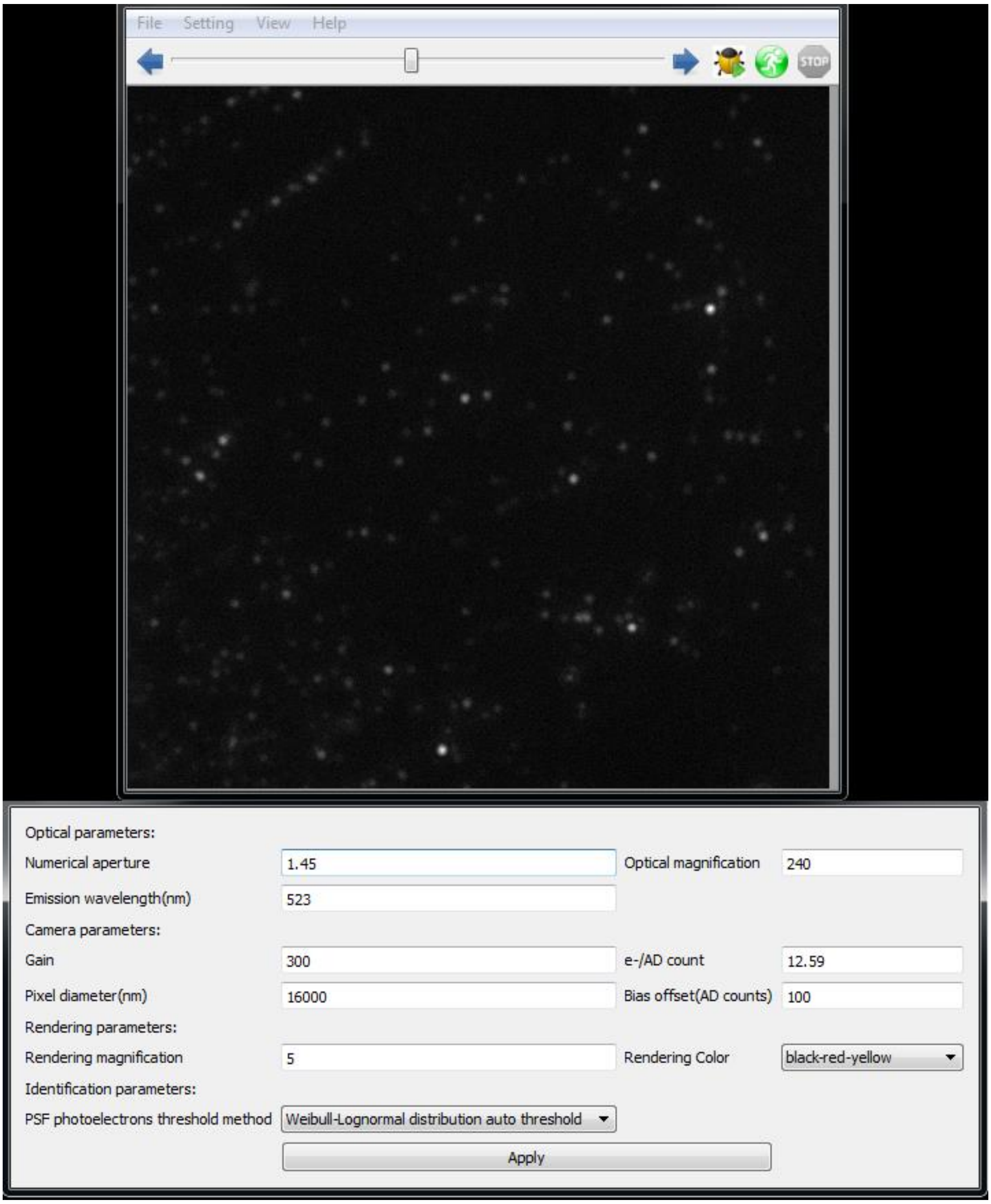

Fig 1. Auto-Bayes graphical user interface.

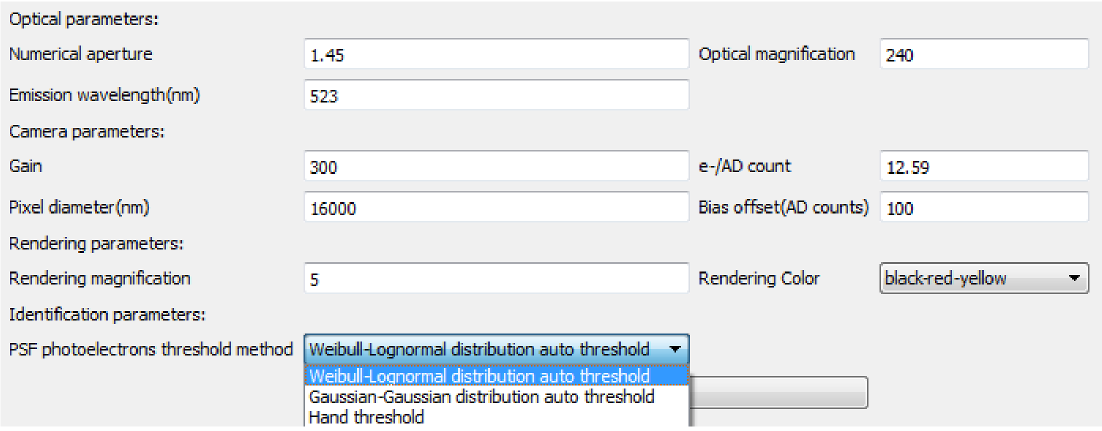

Fig 2. Auto-Bayes thresholding methods.

SENSITIVITY, READOUT NOISE, BASE MEAN LEVEL AND SATURATION LEVEL

A/D Rate — EM = electron multiplication, Con = conventional	Preamp setting	CCD Sensitivity ♦1 (electrons per A/D count)	Single Pixel Noise ♦2 (electrons)	Base Level ♦3 (Counts)
10 MHz 14 bit EM amplifier	1	64.00	99.84	374
	2.4	26.71	65.44	365
	4.8	12.59	53.26	356
5 MHz 14 bit EM amplifier	1	58.00	91.06	355
	2.4	22.79	50.82	350
	4.8	10.62	41.52	345
3 MHz 14 bit EM amplifier	1	57.40	63.71	362
	2.4	22.38	37.15	359
	4.8	10.22	31.48	356
1 MHz 16 bit EM amplifier	1	22.29	38.34	399
	2.4	8.92	23.28	396
	4.8	4.08	19.91	390
3 MHz 14 bit CON amplifier	1	10.61	14.43	368
	2.4	4.17	10.68	366
	4.8	1.87	10.08	357
1 MHz 16 bit CON amplifier	1	3.87	8.67	398
	2.4	1.56	6.82	396
	4.8	0.67	6.06	391
**Saturation Signal per pixel** (MAX horizontal speed 14 bit EM amplifier)	167384		electrons / pixel	

Fig 3. Andor EMCCD performance sheet.
